# What does it mean to be engaged with digital health interventions? A qualitative study into the experiences of engaged users and the views of professionals

**DOI:** 10.1177/20552076241283530

**Published:** 2024-10-03

**Authors:** Saskia M Kelders, Hanneke Kip, Nienke Beerlage-de Jong, Nadine Köhle

**Affiliations:** 1 Department of Health, Psychology and Technology, 3230 University of Twente, Enschede, The Netherlands; 2 648981 Optentia Research Unit, North-West University, Vanderbijlpark, South Africa; 3 Department of Research, Transfore, Deventer, The Netherlands; 4 Section of Health Technology and Services Research, Technical Medical Centre, 3230 University of Twente, Enschede, The Netherlands; 5 Stichting Mindfit, Thubble, Deventer, The Netherlands

**Keywords:** Digital health interventions, engagement, qualitative study, behavioral engagement, cognitive engagement, affective engagement, eHealth

## Abstract

**Objective:**

Digital health interventions (DHIs) hold promise for influencing health behaviors positively, but their widespread implementation and effectiveness remain limited. Engagement is crucial for DHI effectiveness, yet its conceptualization is debated. This qualitative study explores engagement from user and professional perspectives.

**Methods:**

Twenty self-proclaimed engaged health app users participated in semistructured interviews, and 13 professionals working with DHIs completed an online survey.

**Results:**

Interviews with health app users revealed three key components of their sense of engagement: behavioral, cognitive, and affective. Behavioral engagement includes routine, effortless, and dynamic usage; emphasizing the importance of the quality of fit between user and technology over frequency of use. Cognitive engagement encompasses the technology's utility as a tool for supporting behavior change, providing new insights, and enhancing motivation. Affective engagement involves enjoying progress, deriving pleasure from using the technology, and identifying with the technology. Notably, participants exhibited varying emphasis on these components. Professionals, in a parallel inquiry, agreed on the relevance of behavior, cognition, and affect in defining engagement. In their understanding, behavioral engagement is often associated with adherence and frequency of use, while cognitive engagement emphasizes understanding, motivation, and achieving cognitive outcomes. Affective engagement, although diverse, is recognized as a critical dimension. In addition, it was noticeable that users and professionals perceived microengagement (with the DHI) and macroengagement (with the target behavior) as interconnected.

**Conclusion:**

To conclude, this study contributes a nuanced understanding of the multifaceted nature of engagement, informing future measurement of the concept, DHI design, and implementation strategies for improved user experiences and outcomes.

## Introduction

Digital health interventions (DHIs) are a specific form of eHealth. They are a valuable asset for positively influencing health-related behaviors. They have shown to be effective for a wide target group and a wide variety of (mental) health conditions, such as cardiac rehabilitation,^1^ diabetes and obesity,^2^ and depression.^3^ However, in real life, widespread implementation and effectiveness of these interventions is still scarce.^4,5^ Engagement is often mentioned as an important concept that may explain or overcome these issues on implementation and effectiveness.^6–8^ It is posited that a certain degree of engagement of a user with the DHI is necessary for DHIs to be effective^9^ and that more engagement is related to more effectiveness.^7,10,11^ A lack of engagement is therefore seen as a major issue that hampers DHIs reaching their potential effectiveness,^8,12^ especially in DHIs where there is little or no (health care) professional involvement, such as in health apps.^13,14^ If we want to overcome issues with low engagement and accompanying lower effectiveness in DHIs, we should have a good understanding of what the engaging experience is that we need to measure and design for.^6,12,15^ Not only can this insight be used to design more engaging interventions for different target groups, but measuring engagement during the intervention period also opens up the way to continuously adapt interventions to individuals’ needs and context over time.

However, there still is a debate on what engagement to eHealth or DHIs is. A view that has become more prominent in recent years, is that engagement is more than just frequent use of DHIs.^6–8,12^ This marks the difference with a concept as adherence, which is seen as the degree to which DHIs are used as intended by the developers of these interventions.^16,17^ However, there is discussion on what this “more than usage” is. A review on engagement in DHIs described engagement as the extent of usage and a subjective experience characterized by attention, interest, and affect.^7^ Additionally, other researchers stress that there is a difference between microengagement and macroengagement, where microengagement focuses on engagement with the DHI itself, while macroengagement focuses on engagement with the wider intervention goals, that is, the health or behavior change goals.^8^ Our review described engagement to DHIs as a combination of behavior, cognition, and affect, as is common in other fields where engagement is studied.^6^ However, this review acknowledged that the precise content and qualities of the behavioral, cognitive, and affective aspects of engagement to DHI should be further investigated, together with how these components are related to each other and to (health) outcomes. These insights are needed to come to a useful conceptualization of engagement, that can be used to measure the concept and serve as input for design recommendations of DHIs.

What the outcomes of these reviews have in common, is that engagement is, at least partly, shaped by the experience of the people who use DHIs. There have been studies that focus on user's experiences of engagement, for example by reviewing studies on barriers and facilitators for engagement,^18,19^ or on studying the influence of specific features such as gamification on the user experience of engagement.^20,21^ However, most of these studies investigate why some participants did engage with or adhere to a specific DHI, while others did not, thereby focusing on engaging features or predictors of engagement and not on the multifaceted concept of engagement itself. Although this does yield interesting information for the studied DHIs, it is difficult to generalize these findings to other DHIs and to a more encompassing view of what it means to be engaged with a DHI. This last aspect is needed to yield more insight into the content and quality of the components of engagement, as advocated in our earlier review.^6^ To learn more about what it means to be engaged with a DHI, this study focuses on engagement, both directly from the users’ perspective, as well as from the perspective of professionals working with DHIs, for example, mental healthcare professionals and researchers. This last perspective is added to make use of knowledge that exists in the field but has not yet made its way to the slower dissemination path of scientific publications.

The goal of this qualitative study is to gain more insight into how self-proclaimed engaged DHI users (in this case users of health apps, since engagement with these apps is varied^22^), and professionals who work with DHIs, view engagement. This will yield more insight into how to operationalize the different components of engagement (behavior, cognition, and affect) in this context and whether there are any other components to consider. Moreover, this study aims to give insight into the relative importance of the different components of engagement to each other in the context of health apps. Together, these insights might help us gain a better conceptualization of engagement and in that way, in overcoming issues with a lack of engagement and effectiveness of DHIs.

## Methods

For this study, 20 semistructured interviews were conducted with people who are self-proclaimed engaged DHI users, specifically health apps. Health apps were chosen for this study as they are a specific form of DHIs that seem to be able to invoke high engagement in certain people, but not in others.^23^ App stores contain a broad range of apps focused on health and wellbeing, for example fitness apps (often suitable to be used in combination with a wearable), diet-related apps, or mindfulness apps. Some of these apps have been shown to be effective in improving health outcomes, but evidence remains scarce.^24,25^ Although only a small proportion of all available health apps are actually used over a longer period of time, some of these seem to induce high engagement levels in certain users.^23,26^ This makes self-proclaimed engaged health app users an interesting group to learn from what it is that they see as being engaged with a health technology. Additionally, 13 professionals participated in an online survey addressing their views on what engagement to DHIs is.

### Participants

For the interviews, inclusion criteria were being over 18 years of age and feeling engaged with a health app used on a smart phone. As there is still much unclarity about what it means to be engaged with a health app and this is the topic of the current study, we did not objectively assess whether or not potential participants were engaged. Rather, we relied on participants’ own experience as feeling “engaged or in some way involved” with the app, without going into detail what that may mean. Twenty participants were included through convenience sampling. The mean age of the participants was 28 years (ranging from 20 to 60 years) and 13 were female. Twelve participants were university students and the other eight were employed. Five participants used multiple apps with which they were engaged. In total, 19 participants used an app focused on physical health, 8 of which focused on physical activity and diet, 8 focused only on physical activity, 2 on diet only, and 1 on physical activity and sleep. One participant used an app for mental health (Mindfulness). Fifteen participants used an app in combination with a wearable (Fitbit, Garmin, Apple Watch).

For the online survey, professionals who work on or with DHIs (e.g. researchers or psychologists using DHIs for their patients) were invited to participate using the professional networks of the authors. Thirteen professionals with a mean age of 38 years (ranging from 29 to 54 years) participated in the online survey. Of these, seven identified as male and six as female. Most professionals were Dutch (n = 7), two were South African, one Finnish, one Italian, and one did not disclose their nationality. Eight professionals worked in academics, ranging from researcher to associate professor, and five professionals worked in mental health practice as for example, social worker, project leader on eHealth, or clinical psychologist. On average, they indicated having 9.3 years of expertise in their field (ranging from 2 to 17 years). Ten participants considered themselves as experts in DHIs (“somewhat agree” or “strongly agree” with the statement “I’m an expert in eHealth”), eight as experts in psychology, and seven participants considered themselves an expert in Human Computer Interaction.

### Procedure and materials

Interviews with DHI users were held in a quiet space at the participants’ home or at the University of Twente, they were audio recorded and took between 20 and 45 minutes (see also COREQ checklist in Supplemental Appendix 1). Interviews were conducted by four psychology students (see Acknowledgements) under supervision of the first author (SK), who was not present during the interviews. Three of these students identified as female and one as male. All had completed a BSc degree in psychology at the time of data collection and did these interviews as part of their MSc thesis. All students received multiple courses on qualitative research and interviews. Participants for the interviews were recruited through the personal network of the students who conducted the interviews (e.g. using What's app, phone calls, or in person). All participants knew the interviewer before the interview and the student explained that these interviews were conducted for their thesis but within a larger project. The online survey with professionals was made available through an online survey system (Qualtrics) and took between 12 and 60 minutes. Written Informed consent was obtained from participants before the start of the interview, and participants in the online survey provided informed consent within the first page of the online survey. Ethical approval for the study was obtained from the Behavioural Management and Social Sciences, domain Humanities and Social Sciences, ethics committee of the University of Twente (numbers 18223 and 191052).

The semistructured interview scheme for the self-proclaimed engaged users consisted of three major topics to get the participants talking about the health app they use and what that means to them. Prompts were used to let participants explain what they do, think, and feel while using the app. The first topic was focused on which app(s) they were using and with which goals. An example question was “Why did you start using this app?”. The second topic focused on how they use the app in their daily lives. An example question was “Can you explain or show what you usually do with the app?”. The last topic focused on what the app means to them. An example question was “You have indicated that the app is important to you. Can you elaborate on that?”. Questions were deliberately kept open to give participants the opportunity to explain in their own words what it means for them to be engaged to a DHI. The interview scheme was pilot tested and adapted where necessary to make sure that the questions were understood and answerable by participants.

Within the online survey, five open questions were posed on what the professionals considered as engagement to eHealth interventions (see Supplemental Appendix 2). First, a general description of engagement was asked, without prompting any definition or viewpoint in advance. This was done by asking how the professionals would describe engagement to DHIs to a student or client. Second, professionals were asked how they would describe the behavioral, cognitive, and affective components of engagement. These components were introduced in the survey as “behavior (what does the patient do), cognition (what does the patient know and think), and affect (what emotion is the patient experiencing)”. Lastly, professionals were asked for their professional opinion of these components of engagement as to whether these are applicable and together comprise the concept of engagement to DHIs.

### Analysis

Interviews were transcribed verbatim and thematically analyzed using Atlas.ti 9. First, all interviews were read by the first author to familiarize herself with the data. Second, all fragments pertaining to what engagement is to the participants were identified. Next, these fragments were deductively categorized in behavioral, cognitive, and affective engagement. Any fragments that did pertain to engagement, but did not seem to fit into any of the deductive categories remained unlabeled in this first step. Afterwards, all fragments within each component were coded inductively: that is, codes were made from the content of the fragments. As a first step, this was done with 11 interviews and this resulted in 3 coding schemes—1 for each component. These coding schemes were then applied to all fragments, including the previously unlabeled fragments. The last nine interviews did not yield any new codes but gave insight in the content and diversity within the codes. This indicated that data saturation may have been reached.

The first three interviews were coded by two authors (SK and HK), with an initial agreement of 73% (Cohen's kappa = 0.69). All disagreements were discussed until agreement was reached. The code schemes were subsequently adjusted to include the insights from these discussions. The final coding scheme covered the three components behavioral, cognitive, and affective engagement, but additionally included three subcodes for each component. Lastly, an overview was created of how many times each component was coded for each participant.

The answers to the open questions in the online survey for professionals were coded inductively per question as each question covered a different topic (i.e. engagement in general, components of engagement, and applicability and comprehensiveness of components). Inductive analysis was chosen instead of using the code schemes from the interviews with engaged users, to see whether and how the professional view of engagement is different to the user view. First, all answers were read by the first author to familiarize herself with the data. Second, each answer was split into meaningful units which confer a single idea pertaining to the question. Third, each meaningful unit was given an inductive code. After this, the inductive codes were grouped until there was one code scheme for each question. All answers were coded by two authors (SK and HK) and discussed until agreement was reached on the code schemes.

## Results

### Interviews with engaged users

Three hundred and sixty-four fragments that covered what it means to be engaged with a health app for participants were identified. Table 1 gives an overview of the different codes and the number of times each code was found. All participants mentioned all three different components of engagement. Overall, the number of quotes coded in the components behavior (37.6%; n = 137) and cognition (37.1%; n = 135) were similar and somewhat more frequent than quotes relating to affective engagement (25.3%; n = 92). No additional components were identified: any unlabeled fragments from step one of the analysis could be coded within the inductive codes of the three components.

**Table 1.** Overview of used codes in the interviews with engaged users.

Code	Definition	# Quotes (# participants)
Behavior		137 (20)
Routine	Using the DHI (becoming) a routine in daily life and is part of the health behavior	61 (20)
Effortless usage	The DHI is seen as user friendly, and participants do not have to think about how to use it	43 (19)
Dynamic usage	The DHI allows for variation in use, accommodating periods of active use and of nonuse/ moderate use	33 (14)
Cognition		135 (20)
Supportive tool	The participant beliefs that the DHI is supportive in reaching their goals, often related to the DHI giving an overview of collected information	64 (19)
New insight	The participant feels that the DHI provides them with useful information and new insights	37 (16)
Motivation	The participant beliefs that the DHI motivates them in reaching their goals	34 (12)
Affect		92 (20)
Enjoy progress	Enjoying using the DHI because or when it shows the progress you make	34 (16)
Enjoy app	Using the DHI itself is enjoyable and can be addictive	30 (16)
Identity	Feeling a connection with the DHI or missing something when it's not there	28 (15)

DHI: digital health intervention.

#### Behavior

Within the component behavioral engagement, the subcodes “routine” (n = 61, 44.5%), “effortless usage” (n = 43, 31.4%) and “dynamic usage” (n = 33, 24.1%) were found.

##### Routine

Most behavioral engagement quotes were about having a routine with the app and this code was found in every interview. Nineteen participants mentioned that they created a routine with using the app (i.e., using the app at set points during the day and having embedded this in their daily lives): “*I use the app three or four times a day. I use it after breakfast, lunch and dinner and for gaining an overview at the end of the day*” (p. 5). One participant mentioned that he was on the way to create a routine. Participants also mentioned that using the app had become part of their daily lives and part of the behavior they support with the app. For example: “*If I eat something, I type it in*” (p. 20) and “*Running is my hobby but the app is part of it*” (p. 4).

##### Effortless usage

“Effortless usage” was coded second most in the behavioral engagement category and was found in all but one interview. Participants mentioned that using their app is easy and they do not have to think about it. This was seen as part of being engaged with the app. If the app was not user-friendly or would cost more effort, they would stop using it. The following quotes illustrate this: “*But a quick look at it is enough to have everything in your mind. And that is motivating. I think it is clever.*” (p. 8) and: “*You scan a code on your food and then you have automatically the data of it. […] In this way it is really easy, really handy and has a strong relation to the everyday life here. That is a great help and because of this, any other app is out of question for me*.” (p. 18).

##### Dynamic usage

The third code in this category is dynamic usage and was found in 14 interviews. Participants mentioned that while they do feel engaged with the app, there still are periods of active use and of moderate or nonuse. First, when just starting with a new app, participants mentioned that they use it more intensely, for example, “*When I started using the app, I did not take the wearable off. That was so sick! (laughs)*” (p. 3). But when participants got more used to what to expect, usage can become less intensive, but participants still feel engaged, for example, “*When you have been using the app for a while, you learn how many calories a particular meal has. You can go on without being supported by an app*” (p. 5). Changing goals can also influence the intensity of usage, for example, “*Since four months [I use the app] even more intensely, because my motivation changed. Before, it was important to have an overview over my results, but it was not that important to reach my goals every day. (…) At the moment I keep my results and goals in mind and monitor my behavior regularly*” (p. 6). Lastly, participants also mention a risk of dynamic usage, namely that it can be easy to slip into an unwanted nonuse pattern after a few days of nonuse: “*If I, if you take a break from two days then it is no longer in your rhythm. And then you take a longer break. I realize it quickly, that you do not think about it*.” (p. 11).

#### Cognition

Within the component of cognitive engagement, the subcodes supportive tool (n = 64, 47.4%), new insights (n = 37, 27.4%) and motivation (n = 34, 25.2%) were found.

##### Supportive tool

The subcode supportive tool is mentioned most often in this category, and by almost all participants (19 out of 20). This code describes that participants belief the app supports them in achieving their goals, by doing things they cannot do themselves, or that the app does it better than they could have done by themselves, which means that “*both devices [app and wearable] support me and it's all about being more active*” (p. 1). This novel way of supporting behavior change is often related to collecting and presenting data on, for example, the number of steps participants have taken, or the number of calories burned since this provides them with new knowledge that is hard to gain without the use of the app. For some participants, this overview helps them gain a sense of control on their behavior. For example, “*What [does the app] mean to me? Yes, it is a nice gadget. It is not essential, that I could not live without it, but it offers a special control and overview over my habits that I want to correct. Yeah, that is in some ways pretty important*” (p. 17). For others, the overview's benefit lies in gaining confirmation of what that have done, for example, “*It is exactly shown how often you meditated, how many sessions you completed the average time you meditated*.” (p. 11). When this overview is lost, participants can experience frustration: “*I need the confirmation that I really did the run. Some time ago, the app did not track my run. I was really upset and considered doing it again just in order to have the confirmation that I did it*.” (p. 4). Lastly, for some, having this supportive tool also entails commitment, or putting in effort themselves, for example, in working with the app or making sense of the collected data. This is not something that seems to bother them, it is part of how they are engaged. Participants described, for example, that they spend quite some time entering data: “*(…) It takes some time to track a meal but I think it is worth it. Other people often do not understand that*.” (p. 5).

##### New insights

The second most used code in the cognitive engagement category is new insights. This code was mentioned by 16 participants and is about being engaged with the app because of the new insight the app gives them based on the information that is provided by the app. As participant 12 puts it: “*It just gives me insight, and that is the most important thing to me*.” (p. 12). Participants explain that the information itself is interesting to them, but that they also use the information to draw conclusions on how they are doing in relation to their goals. They, for example, compare their data from different times to gain insight in their progress (e.g., “*I like that I can compare my current data with the data from the past*.” (p. 1)), or use the information to adjust their plans on how to achieve their goals: “*And I also count my calories and then I adapt my eating behavior*.” (p. 10). Compared to the subcode supportive tool, fragments with the code insight are more related to giving meaning to the data, while the fragments with the code supportive tool are about that the app collects or shows data.

##### Motivation

The last subcode within cognitive engagement is motivation. This code is mentioned by 12 participants and is about that the app increases their motivation for the targeted health behavior. The source of the motivation can be the app itself, the data the app collects, or the insight that participants gain from that. The most important aspect within this subcode is that participants specifically mention the motivation that comes with this. Examples are participant 6 who indicates “*Well, it's my motivator to do more sport. It's motivating me every day new*.” (p. 6), or participant 4 who states, “*I would not be able to motivate myself without the app*” (p. 4)*.* For some, the messages or reminders during the day are the most important motivator (e.g. “*[the app says] ‘you’re doing great!’, and that literally motivates!*” (p. 13), but for others, it is the more long-term overview of what they have done that motivates them: “*I think the app is important as it is motivating. The app motivates me by creating graphics in which current personal data is compared to earlier entries*” (p. 1).

#### Affect

Within the component of affective engagement, the subcodes “enjoyment of progress” (n = 34, 37.0%), “enjoyment of the app” (n = 30, 32.6%) and “identity” (n = 28, 30.4%) were found almost equally often.

##### Enjoyment of Progress

Enjoy progress was mentioned by 16 participants who explained that they enjoy achieving their goals supported by the app. They stated that the app makes them feel satisfied, proud or happy by showing that they are achieving their own goals, for example, by complimenting them or displaying fireworks: “*I like the special effects when achieving my goal. I enjoy them*” (p. 3) or “*Yeah, if you reach 10,000 steps, you get fireworks!*” (p. 15). It also seems to make them enjoy the target behavior more, for example, “*Using the app is not a hobby, no. But it makes my hobby more enjoyable*.” (p. 5). However, there is another side to this code as participants can feel frustrated when not reaching their goals (e.g. “*It is fun, except when you stop exercising*” (p. 3)), or not even use the app when they know it is likely that they will not achieve their goals.

##### Enjoyment of the App

Sixteen participants mentioned that they enjoy using the app itself. Participants described that using the app itself is “*just fun*” (p. 16) or “*it is cool!*” (p. 19). Some participants go further into saying “*I love using my watch!*” (p. 10) and three participants note that they feel or have felt addicted to the app. There seems to be an inter-relationship between addiction to the app and to the behavior which participants do not seem to differentiate between as can be seen in the following quote: “*First, I thought that I would use the app and the wearable for only a few days, but now I am addicted. I always want to achieve my goal of taking 10.000 steps now*.” (p. 2).

##### Identity

Identity is mentioned by 15 participants. Most participants mention that they feel a connection with the app and that it fits them as a person. They state that they “*would miss something if it was not there anymore*” (p. 2), or that the app is their “buddy”: “*It means a lot to me, for sports. It is and will be my buddy. I can’t do without my watch*” (p. 15). They state that the app is an important part of their life, but it is “*of course not the elixir of life!*” (p. 7). Some participants seem to go further in identifying with the app, for example, “*I would not exchange it against another app. I want to use this specific one*.” (p. 6) and even “*I cannot live without it, I think. I am in a really bad mood when I cannot wear it*.” (p. 10).

#### Salience of components in individuals

To explore whether there are differences in the salience of the components behavioral, cognitive, and affective engagement for individuals, an overview of the proportion of quotes from each interview relating to each of the components of engagement was made (see Table 2). From the table it can be seen that all components of engagement are visible in all participants, but the percentages range from 11 to 61, indicating that there is some variance in the salience of components between individuals.

**Table 2.** Individual distribution of quotes of engaged users over the components.

Participant	% Behavior	% Cognition	% Affect
1	21	43	36
2	50	20	30
3	39	31	31
4	27	53	20
5	43	43	14
6	29	55	16
7	41	29	29
8	58	16	26
9	50	21	29
10	25	35	40
11	39	31	31
12	31	31	38
13	35	45	20
14	40	33	27
15	29	57	14
16	25	40	35
17	23	54	23
18	61	28	11
19	46	36	18
20	56	33	11
Total	**38**	**37**	**25**

Note: White is 0–20%; light green 21–40%; green 41–60%; and dark green >60%.

Eight participants mentioned behavioral engagement most often (between 40% and 61%). They seem to use the app quite routinely and it does not cost them much effort, but they do not have really strong feelings or thoughts about it. Interestingly, two of these participants mentioned that they have started using the app because they have the wearable and it is expensive. This was not found in any of the other interviews. Seven participants mentioned cognitive engagement most often (between 40% and 57%). For these participants, the app seems to be mostly a means to achieve a goal: they know the app can enhance their motivation and ability to reach their goals and that makes it important to them. In three participants, all categories are mentioned relatively evenly (between 30% and 40%), seemingly making them engaged on all levels. One participant scored relatively high on both behavioral and cognitive engagement (both 43%, p. 5). For this participant, emotions do not seem to play a real role in engaging with the app. Lastly, one participant mentioned affective engagement most often (40%, p. 10). They really enjoyed (even loved) using the app and felt connected to it, which seems to be their form of engagement.

### Professional view on engagement

#### Engagement in general

From the answers to the question how the professionals would describe engagement to a student or patient, 24 fragments were coded. Of these, three fragments from three different professionals were coded as not pertaining to engagement as they described actual DHIs, and not engagement to DHIs. The remaining 21 fragments stemmed from the interviews of 10 different professionals. Two participants mentioned the components behavior, cognition, and affect without being prompted. Other participants described their view on engagement in general terms, conveying often one main dimension (n = 5), or describe multiple related dimensions. The most often mentioned code was “**usage**” (n = 4) in which (part of) engagement was related to how often a DHI was used, expressed, for example, as “*engagement is strong when the patient uses the intervention on regular basis, preferably every day*.” (exp12). Three professionals stressed that engagement was closely “**goal-related**” (n = 3), for example, “*It relates to patients and caregivers productively using the technology to achieve previously set goals in everyday health management of chronic conditions*.” (exp10). “**Motivation**,” “**understanding**,” and “**involvement**” were all mentioned by two professionals to be important aspects of engagement. Involvement was often used as a general description of engagement (e.g., “*[engagement is] the extent to which people are involved in an intervention*” (exp7), while motivation and understanding are mentioned as things that engaged users are likely to experience (e.g., “*Participants who are fully engaged in a task will experience a high level of motivation to continue to engage with the task at hand*.” (exp5)). Other codes that were mentioned in describing engagement by one professional are: bond, curiosity, enjoyment, a positive attitude, a rich user experience, openness to new experiences, and a willingness to use eHealth technology.

#### Components of engagement

Table 3 presents the codes used to describe behavioral, cognitive and affective engagement by the 13 professionals. Most professionals view behavioral engagement as related to the use of the DHI, either participants using the intervention as intended by the developers or prescribers of the intervention (n = 5) or participants using the intervention (more) frequently (n = 3). Some professionals relate behavioral engagement to behavioral outcomes of the intervention (n = 3), for example, “*they come to appointments more often, can reach out more easily, and have more contact with counselors that way*” (exp2). Other professionals seem to refer to a certain quality of behavior that is seen as engaged: attention or interest while using the technology (n = 3), openness to using the intervention (n = 2), or putting in effort in using the technology (n = 1).

**Table 3.** Overview of used codes by the professionals in the questionnaire.

Behavior	# Fragments (# professionals)	Cognition	# Fragments (# professionals)	Affect	# Fragments (# professionals)
Use as intended	5 (5)	Cognitive outcomes	6 (5)	Emotional connection	2 (2)
Frequent use	3 (3)	Added value	3 (3)	Emotions	2 (2)
Behavioral outcomes	3 (3)	Motivation	3 (3)	Positive attitude	2 (2)
Attention/interest	3 (2)	Mental effort	3 (3)	Positive emotions	2 (2)
Openness	2 (2)	Attention/interest	2 (2)	Pride	1 (1)
Effort	1 (1)	Understanding	2 (2)	Support	1 (1)
		Right challenge	1 (1)	Trust	1 (1)
				Comfortable using	1 (1)
				Enjoy intervention	1 (1)
				Enjoy progress	1 (1)
				Involvement	1 (1)
				Loyalty	1 (1)
				Motivation	1 (1)
				No distinction between cognition and affect	1 (1)
**Total**	**17**	**Total**	**20**	**Total**	**18**

Experiencing cognitive outcomes are most often mentioned as being part of cognitive engagement (n = 5). An example is*:* “*They feel they are learning something new, and they are changing behavior based on this*” (exp12). Other professionals mention that cognitive engagement means that participants see the added value of the technology (n = 3), or are motivated (n = 3) to use the intervention or to perform the goal behavior of the intervention. Lastly, professionals mention different aspects related to how people use or experience the use of the DHI as part of cognitive engagement. Examples include putting in mental effort while using the technology (n = 3), understanding how to use the technology (n = 2), or experiencing that the technology provides the right level of challenge (n = 1).

The views on what affective engagement entails seem more diverse than the views on behavioral and cognitive engagement, with a total of 14 different codes. Professionals vary in their views on what kind of emotions make up affective engagement. Some mention “emotions” (n = 2) or an “emotional connection” (n = 2) as a broad view of affective engagement, while others are more specific in adding that these emotions or attitudes should be positive (both n = 2). Others mention specific emotions or feelings as “pride” or being “comfortable using” the intervention (both n = 1). Enjoyment is mentioned twice as a specific emotion. Similar to the interviews with users of DHIs, it was mentioned (n = 1) as enjoying to use the intervention and (n = 1) as enjoying the progress a participant can experience with the intervention. Lastly, one professional mentioned that it might not be “*always so useful*” to make a distinction between cognitive and affective engagement as “*having a dedicated intention for sustained use has a dynamic intertwined cognitive and affective aspect*” (exp11).

On the question whether describing engagement as consisting of the components behavior, cognition, and affect is applicable to eHealth interventions all 13 professionals answered positively. A few remarks were made stating that it might be that there are other aspects in play as well (n = 1), and that it remains important not to confuse engagement to eHealth technology with other forms of engagement as patient engagement (n = 1).

## Discussion

### Principal results

This study aimed to give more insight into both the experience of engagement in users of DHIs—in this case health apps— and professionals’ views on what engagement is. In both groups, we found support for the previously established view of engagement consisting of behavior, cognition, and affect.^6^ Moreover, this study has yielded more insight into the qualities of engaged behavior, cognition, and affect. Based on the interviews with self-proclaimed engaged health app users, it appeared that all participants were in some way and to some extent engaged behaviorally, cognitively, and affectively. In this study, we found that for being behaviorally engaged, routine is important as well as having to spend little to no effort in using the technology. Moreover, use is dynamic: there are active periods in which participants use a technology more intensively and periods of moderate or even nonuse. However, having these periods of moderate or nonuse in itself does not mean participants are not engaged anymore. Rather, this seems to be part of the process of engagement. Being cognitively engaged consists of users knowing that the technology is a supportive tool that can help them achieve their goals. Moreover, the technology gives new insights and motivates users to achieve their goals. Affective engagement consists of users experiencing positive emotions in working with the technology itself and in the progress that they achieve with the technology. Moreover, affectively engaged users identify with the technology in some way.

In this study, behavioral engagement seemed to be more about having a routine in using the technology and making it a part of your life, than about the frequency of using it, as is often used as a measure of engagement in DHIs.^7^ Participants seemed to focus more on incorporating it in routines since the frequency of using an app is much more dependent on what technology you use, with what goals, and in what phase of (dynamic) use you are. This is similar to the argument made in the paper of Sieverink et al. on that adherence is more than just the frequency of use.^17^ For adherence, it is not “the more use of a technology, the better,” but it's about adhering to the intended use that corresponds to the goals you want to achieve. Behavioral engagement seems to be even less about the frequency of use than adherence: the ability of an individual to create a routine with a DHI, and to adapt this routine based on their current goals and progress, seems to be an important part of behavioral engagement. That behavioral engagement itself is already more than simply using the technology as often as possible, mirrors insights in different fields such as work engagement. In work engagement, “vigor” can be seen as behavioral engagement.^27^ But this is not just seen as showing up for work, but also as the quality of the behavior, in this case working with a high level of energy, putting in effort and showing persistence even when facing difficulties.^27^ For engagement with DHIs, in this case using a health app, the quality of the behavior might be (1) making it a routine, (2) using without needing to spend effort, and (3) being able to adapt the routine to your own goals. This entails that it is important to distinguish concepts as (behavioral) engagement, adherence, and usage, and that for example, usage cannot be employed to measure engagement, as is often still done.^18,28^

Cognitive engagement with a DHI was found to be prominently related to the goals of the users. People have to have the belief that the technology is a supportive tool, that, by for example, giving an overview of their behavior, can give new insights and motivation to achieve their goals. This connection to personal goals is related to the importance of personal relevance to engagement as stated in earlier studies on DHIs.^11,29,30^ The aspect of new insights also has some similarity to the “attention and interest” part of the definition of engagement of Perski et al.,^7^ in the sense that participants can only achieve this insight when they pay attention to the DHI and are at least somewhat interested in it. However, the current study suggests cognitive engagement is broader than suggested in other definitions of DHI engagement.^7,31^ Interesting to note is that while cognitively engaged, participants were willing to put in mental effort as long as it helps them reach their goals. This is somewhat conflicting with being behaviorally engaged, which is enhanced by not needing to put in effort. It seems that although technology should make things easier, if people think technology can be supportive for them, they are willing to spend time and energy to gain most out of it. When designing for engagement, it seems to be useful to keep this in mind and design DHIs in such a way that the users are guided to and are given the opportunity to spend their mental effort at the most useful point, for example, where there is a need for reflection on their earlier behavior. However, it might be beneficial to make this mental effort optional to also cater to individuals who are more behaviorally engaged.

Lastly, this study found that affective engagement played a role for every participant, but was less salient in most participants. Interestingly, affect was not only focused on the technology itself, but also on achieving goals, so it seems to be more than just user-engagement.^31^ Second, although most participants mentioned positive affect when achieving goals, negative affect also seemed to play a role. Participants experienced frustration when not achieving their goals and for some this may enhance their motivation to go on. However, as it is important to make sure goals are achievable,^32^ it seems important to limit the number of times people do not achieve their goals or adapt the way this is communicated. Nonetheless, it might be interesting to see if there is a way to harness this negative affect or even use aversive feedback to increase the persuasive power of a DHI.^33^ Lastly, identity seemed to play a role in affective engagement: users need to be able to identify in some way with the technology and what it stands for. Within, for example, mental health, this might pose a challenge as many people might not want to identify themselves with issues such as depression or anxiety. However, framing a technology in more positive terms (e.g. using positive psychology to focus on strengths to overcome depressive feelings) might help overcome this issue.^34^

The results of the professional views on engagement mostly corroborated earlier studies (e.g. ^6,7,35^) and the results of the interviews with health app users. First, the definitions of engagement given by the professionals varied widely, resonating that there is still a lot of debate regarding the precise definition of engagement in literature.^6,12^ Second, usage was the most often mentioned aspect of engagement by the professionals, which seems to be related to the issue that the frequency of use is still often used to measure engagement, whereas the view that engagement is more than usage is becoming more prominent and also corroborated by the results of interviews with engaged users in this study.^6,8,12^ When prompted with the components behavior, cognition, and affect, all professionals agreed that these are relevant. However, when asked about what these components entail, a diffuse image comes forward, showing that more insight in what the concepts entail is needed. Interestingly, in specifying these components, not only behaviors or cognitions related to the DHI itself, but also outcomes of being engaged with a DHI were often mentioned. This might show that professionals tend to see engagement with a DHI and with the target behavior as a singular- or at least complementary concepts, which was also how engaged users in this study seemed to view engagement.

### Concept of engagement

Together, behavior, cognition, and affect seem to be the core building blocks that shape individual engagement. From this study, we have seen that these building blocks can have different sizes and importance in different individuals. It seems that these differently sized blocks shape an individual's engagement: the blocks can be larger or smaller and they can be arranged in a different way so that the blocks support each other in different ways. We do not yet know what influences how users shape their own engagement, but this would be interesting to explore in future research. In the context of DHIs, we can hypothesize that having at least some of each component of engagement is beneficial. For example, it might be that for one individual behavioral engagement is largest: they have created a routine with it, which costs them little effort, but there are periods in which they slip from their routine. If this person also has some cognitive or affective engagement, this might be enough to re-engage them and end a nonactive period. They might realize that they know the app enhances their ability to achieve their goals, or they might miss the enjoyment of seeing that they reached their goal within the app. This is similar to the description of the stages of engagement from O’Brien et al.,^31^ where it is suggested that there are different stages of engagement (e.g., point of engagement, disengagement, and re-engagement) and during these stages, different aspects are important. However, from the current study, it cannot be said that there is a best “shape” of engagement or a “usual way” of getting re-engaged (as proposed by O’Brien and Toms^31^), as this seems to highly vary between individuals. Furthermore, the shape of engagement might also depend on the target behavior. For example, in medication adherence apps, it might be enough to just be behaviorally engaged, because this is mainly about performing a desired behavior. However, this might pose a danger in a period of less engagement as little is done to get people to re-engage. Cognitive engagement might be more important in, for example, mental health interventions where people are expected to reflect on themselves and thus put in mental effort; and affective engagement might be more important for people who cannot, or do not want to, rely on cognitive abilities primarily. All in all, it seems important for the technology to allow and design for the different forms of engagement to make sure users will not be dependent on one form or shape of engagement that does not fit their own engagement style.

Another interesting finding from this study is that, for users and professionals, being engaged with an app (microengagement) and with a target behavior (macroengagement) are strongly related and it seems that they cannot be understood as separate entities. This is seen in behavioral engagement, where routine is important, but this is both a routine with using the technology as with performing the target behavior. In cognitive engagement, this is seen in the close link between the technology and the goals it is used for, and in affective engagement this is seen in the importance of enjoying both the technology and the target behavior. This finding resonates with other studies that have indicated that within DHIs, emphasis should be on fostering engagement with the treatment and not on engagement with the technology,^35,36^ or that engagement with the technology and with the target behavior should both be considered.^8^ The current study supports those views in that it once again indicates that it is important to consider engagement with the target behavior (or treatment). Moreover, it adds that in practice the two types of engagement cannot be viewed separately. For engaged users, they are so intertwined that they are often viewed as one thing. In theory, it would be great if people use an app to change their behavior and get so engaged with the new behavior that they don't need the app anymore. However, it might be very helpful to also induce engagement with the app, as this can provide a method for re-engagement with the behavior when this is needed. So, in this context, designing for engagement should, in our view, always focus on the intertwined engagement with the technology and the target behavior.

### Limitations and future work

Our goal was to provide insight into the experience of engagement with DHIs. For that purpose, we chose to interview users of health apps. Almost all of the included participants used a health app focused on physical health (activity and diet), often in combination with a wearable. Our results should be interpreted within this context, especially considering the intertwined relationship between target behavior and engagement; it might be that some codes we found are specific to users of this kind of apps which have certain specific characteristics. First, this kind of health apps are often only briefly used each time: for example, a quick view to see how many steps have already been taken. Attributes as challenge or esthetics that have been found to be important to user-engagement in general,^31^ might not be that important in this context, but may play a role in DHIs that rely on longer interactions, such as completing modules in an eMental health intervention.^37^ However, this might make our finding that enjoying the use of the technology is important more striking: if it is already important in brief interactions, it may be even more important in longer interactions. Second, the finding that enjoying progress is an important part of being affectively engaged, might be specific for apps focusing on physical activity, especially in combination with a wearable. In this context, progress can be monitored and seen all the time and there are specific goals that you can reach every day (e.g., a certain number of steps). In other areas it might be more difficult to show progress or achieving goals daily, as for example, in mental health there might not be progress every day and in chronic disease management the focus is even on stability rather than progress. However, as enjoying progress can be an important part of engagement, these fields might employ, for example, principles from gamification or goalsetting to show daily progress and thereby enhance engagement.^38–40^

A last limitation is that although in this paper we have presented the components of engagement as separate from each other, it seems to be not as clear cut in reality, as we have seen that the components can influence each other, or even blend into each other. For example, technology as a supportive tool (or a lack thereof) is part of cognitive engagement, but can also induce strong emotions (affective engagement). Or for example, routine (behavioral engagement) might only be established after a user has been cognitively and affectively engaged for a while. The idea that the components of engagement are not mutually exclusive and influence each other is also seen in different fields, for example, in student-engagement where the dynamic interplay between the components is seen as crucial to understanding engagement.^41^

Based on the results of this study, future work should validate these findings in other DHI-related contexts. Furthermore, it seems essential that there is a validated way of measuring the different components of engagement to DHIs. This would allow research to step away from measuring usage of a system or self-proclaimed engagement as a proxy for actual engagement and towards objectively measuring the full concept of engagement. This could lead to much more insight into different levels and shapes of engagement and would provide a better starting point to create more engaging eHealth technologies. The results of this study have already served as a starting point for creating such a validated measure of engagement, namely the TWente Engagement with Ehealth Technologies Scale (TWEETS).^42^

## Conclusions

To conclude, this study provides evidence that behavior, cognition, and affect are the building blocks that shape individual engagement. We have shown that routine, effortless, and dynamic usage are important when it comes to behavioral engagement, which stresses that engagement should be seen and measured as something other than usage of a system. Cognitive engagement consists of technology as a supportive tool, new insights, and motivation, which shows that engagement should always be seen in the context of the goals of the users. Affective engagement is seen as a combination of enjoying the technology, enjoying progress, and identity, which indicates that creating a pleasurable user-experience is part of the key to designing engaging eHealth technologies, but that it is not the whole story. Overall, we can conclude that engagement is a multifaceted concept with interrelated components, and measurement should strive to integrate all different aspects instead. More research is needed on the interrelatedness of (components of) engagement, design, and effectiveness of DHIs.

## Supplemental Material

sj-docx-1-dhj-10.1177_20552076241283530 - Supplemental material for What does it mean to be engaged with digital health interventions? A qualitative study into the experiences of engaged users and the views of professionals Supplemental material, sj-docx-1-dhj-10.1177_20552076241283530 for What does it mean to be engaged with digital health interventions? A qualitative study into the experiences of engaged users and the views of professionals by Saskia M Kelders, Hanneke Kip, Nienke Beerlage-de Jong and Nadine Köhle in DIGITAL HEALTH

sj-docx-2-dhj-10.1177_20552076241283530 - Supplemental material for What does it mean to be engaged with digital health interventions? A qualitative study into the experiences of engaged users and the views of professionals Supplemental material, sj-docx-2-dhj-10.1177_20552076241283530 for What does it mean to be engaged with digital health interventions? A qualitative study into the experiences of engaged users and the views of professionals by Saskia M Kelders, Hanneke Kip, Nienke Beerlage-de Jong and Nadine Köhle in DIGITAL HEALTH
