# Supplementary material for: What does it mean to be engaged with digital health interventions? A qualitative study into the experiences of engaged users and the views of professionals
Source: Digit Health. 2024 Oct 3;10:20552076241283530. doi: 10.1177/20552076241283530 (PMC11457276; doi:10.1177/20552076241283530)
Supplement: sj-docx-2-dhj-10.1177_20552076241283530 - Supplemental material for What does it mean to be engaged with digital health interventions? A qualitative study into the experiences of engaged users and the views of professionals [file sj-docx-2-dhj-10.1177_20552076241283530.docx]

What is your gender?

o Male

o Female

o Other

What age are you?

What is your nationality?

o Dutch

o German

o South African

o Other:

What is your occupation?

I'm an expert in the field of:

Strongly agree (13)

Somewhat agree (14)

Neither agree nor disagree (15)

Somewhat Strongly disagree (16) disagree (17)

eHealth (1)

Human Computer Interaction (2)

Psychology (3)

o o

o

o o

o

o o

o

o o

o

o o

o

How would you describe your field of expertise? For instance, is there any specific target group you are working with.

How many years of experience do you have in your field of expertise?

How would you explain what engagement to eHealth technology is (e.g. to a student or non- professional)?

In the scientific literature, engagement is often seen as consisting of behavior (what does the patient do), cognition (what does the patient know and think) and affect (what emotion is the patient experiencing).

If you think of behavioral engagement to eHealth technologies, what would that be?

If you think of cognitive engagement to eHealth technologies, what would that be?

If you think of affective engagement to eHealth technologies, what would that be?

In how far do you think this way of describing engagement is applicable to eHealth?
